# Assessing Proactive Language Control: Does Predictability of Language Sequences Benefit Language Switching?

**DOI:** 10.5334/joc.219

**Published:** 2022-04-11

**Authors:** Tanja C. Roembke, Andrea M. Philipp, Iring Koch

**Affiliations:** 1 Chair of Cognitive and Experimental Psychology, Institute of Psychology, RWTH Aachen University, DE

**Keywords:** Bilingualism, proactive language control, sequence predictability, language switching

## Abstract

Multilinguals often switch between the languages they speak. One open question is to what extent they can use anticipatory—or proactive—language control to reduce interference from non-target languages during language switching. In three experiments, unbalanced German-English bilinguals (N_1_ = 24; N_2_ = 35; N_3_ = 37) named pictures in their L1 or L2 in mixed blocks. In all but the penultimate block, the language sequence in which pictures were named was predictable (e.g., L1, L1, L2, L2, etc.), thus allowing participants to prepare for upcoming trials. Performance in the non-predictable block was compared to average performance in predictable sequence blocks right before and after, thus controlling for practice effects. We predicted that language switching would be facilitated during predictable language trials, indicative of proactive language control. However, for Experiments 1–2, there was no evidence for a predictability benefit across both experiments. When the number of items that had to be switched between was reduced to two (Experiment 3), a limited repetition-specific predictability effect emerged. These findings suggest that people do not use preparatory processes endogenously on the basis of regularities in the language sequence to reduce interference during language switching, unless the specific item that needs to be produced can be anticipated.

Up to 50% of the world population is considered bilingual, though estimates may range significantly depending on country and continent (Luk, 2017). Even as bilingualism is generally considered an advantage (e.g., on the global labor market), it may also include some unique challenges. For example, during bilingual language processing, words from the non-target language are also activated (e.g., Hermans, Bongaerts, De Bot, & Schreuder, 1998; Meade, Midgley, Dijkstra, & Holcomb, 2018). Such competition may be particularly strong when switching between languages (particularly if switching is the result of external constraints; Blanco-Elorrieta & Pylkkänen, 2017, 2018), as it, by definition, is limited to situations where a previous target language becomes no longer relevant. Language switching can happen both in language comprehension (e.g., when hearing a song with English lyrics on a German radio show) as well as production (e.g., when talking to a friend in German and then singing along to an English song). We do not currently fully understand how such switching between languages occurs.

Switching between languages in language production is thought to be supported by language control, the process by which one guarantees that language production occurs in the target language (Declerck et al., 2017). In the current study, we set out to investigate how language switching is influenced by preparatory processes in three experiments. While previous research suggests that such processes can facilitate language switching at least under some circumstances, it is currently unclear whether this is possible as a result of sequence regularity. Thus, we asked if language switching was facilitated when it occurred in predictable language sequences versus random ones. Our study will give insights into the extent to which people can implement anticipatory—so-called proactive—language control to minimize interference when accessing different languages on the basis of statistical regularities (i.e., predictable language sequences).

## The role of proactive language control in bilingual language processing

One can distinguish between at least two types of language control: reactive and proactive language control (Declerck, 2020; Ma et al., 2016; Peeters & Dijkstra, 2018). Reactive language control is thought to be transient and the result of cross-language competition during response selection. As such, it is thought to be more often driven by exogenous cues like a stimulus picture that has to be named in a specific language. In contrast, proactive language control is defined as more sustained language control that is implemented when *expecting* that cross-language competition will occur (Declerck, 2020). It is therefore more endogenous. Even as proactive language control is a feature of many models of language control, research has been more focused on the reactive kind (Green, 1998), thus leaving open to what extent proactive language control is operative during bilingual language processing (Declerck, 2020; Declerck et al., 2015).

Both types of language control can be measured in a language switching paradigm: During a typical language switching experiment, bilingual participants name a picture or digit in one of their two languages (L1, L2) on the basis of a language cue (e.g., Christoffels et al., 2007; Declerck et al., 2017; Meuter & Allport, 1999). In this paradigm, there are two possible language transitions: repetition trials, where the same language is used as in the previous trial, and switch trials, where a different language was used on a previous trial than on the current one. In general, naming in switch trials tends to be slower and less accurate than in repetition trials, called switch costs. Moreover, the switching paradigm can be paired with pure language blocks, where only one language has to be used. Similar to switch costs, performance is worse (slower, less accurate) for both languages on mixed than pure language blocks, called mixing costs. In addition to the mixing costs, it is also often observed that L1 is more affected by mixing than L2 to the extent that naming in L1 becomes even slower than naming in L2 in mixed language blocks, even though the opposite was true in pure language blocks (Christoffels et al., 2007; Jylkkä et al., 2018). This phenomenon is referred to as L1 slowing or reversed language dominance effect (see Gade et al., 2021). Interestingly, performance costs related to language switching are reduced or even non-existent when participants are allowed to switch voluntarily, suggesting that language switching outside the laboratory (where voluntary switching may be more common) may be less effortful than cued language switching suggests (Blanco-Elorrieta & Pylkkänen, 2017, 2018).

Switch costs have been hypothesized to be the result of reactive language control: In one of the most influential models of language control, the inhibitory control model (ICM; Green, 1998), inhibition is proportional to the amount of language competition (i.e., interference) caused by the non-dominant language. As a result, it predicts that more inhibition is needed for suppressing L1, the typically more dominant language that has been acquired first and/or is used more frequently than L2 (or L3, etc. in multilinguals). These asymmetric switch costs are sometimes observed (e.g., Experiment 1 by Costa & Santesteban, 2004; Meuter & Allport, 1999; though not always, see Experiments 2–4 by Costa & Santesteban, 2004; Declerck et al., 2012; Gade et al., 2021; Slevc et al., 2016) and are consistent with the ICM predictions.

In contrast, proactive language control is thought to be reflected in mixing costs and in L1 slowing (Declerck, 2020). Mixing costs hereby reflect the costs that emerge due to the general readiness of responding in either L1 or L2 in mixed language blocks (whereas only one language is relevant in pure language blocks). Additionally, according to the ICM (Green, 1998), the more dominant L1 is inhibited proactively with the help of language tags that are attached to each word representation in order to avoid premature responses of the dominant (i.e. higher activated) language (Declerck et al., 2013), thus leading to the slowing of L1 production. This indeed reflects a sustained proactive control, as the inhibition of L1 in this case lasts across multiple trials or even blocks of trials, while the reactive inhibitory control affects performance on a trial-to-trial basis.

## Testing proactive language control: Preparation time and predictability

There is a frequent alternative to investigate proactive language control by manipulating the amount of time the language is cued in advance during mixed (switching) blocks: If the cueing period is longer, preparatory processes may be used to inhibit the non-target language or increase activation of the target language to reduce the expected between-language competition. In contrast, if the cue is presented only right before the to-be-named picture/digit becomes visible or even simultaneously, no such processes may be implemented. Findings using this manipulation of preparation time were not fully consistent: Manipulating the cuing interval, Philipp, Gade, and Koch (2007) surprisingly found an increase in switch costs for longer preparation times (Experiment 1). However, more recent studies have reported evidence for proactive language control using this paradigm, where longer preparation intervals indeed led to an overall faster performance (e.g., Fink & Goldrick, 2015; Verhoef et al., 2009) and lower switch costs (e.g., Fink & Goldrick, 2015; Mosca & Clahsen, 2016). Interestingly, Costa and Santesteban (2004) found that while longer preparation times reduced switch costs, they did not impact L1 slowing. Nevertheless, together, these results have been interpreted to suggest that proactive language control can be implemented when preparation time is longer (e.g., 800 ms; Mosca & Clahsen, 2016).

Preparatory processes can also be investigated based on manipulations of predictability. For example, this was done in a study by Declerck et al. (2015) (see also Declerck et al., 2013): In their study, unbalanced bilingual participants switched in double alternations (e.g., AABB) between two languages in over-trained word sequences (e.g., the days of the week) or other word sequences. Thus, it was manipulated whether it was possible to predict the language and/or the exact upcoming concept: For example, when naming weekdays in a predictable language sequence (e.g., Montag (Monday), Dienstag (Tuesday), Wednesday, Thursday, etc.), one knows both what day (e.g., Friday) and what language (e.g., German) one needs to produce it in. Performance could then be compared to when only one of these two factors was known. This design allowed them to estimate how much people benefitted from knowing the exact semantic concept and language, respectively. The authors found a general predictability benefit of 43 ms when only the language of an upcoming trial, but not the concept, was known (Experiment 3). Yet, in the same experiment, knowing only the language but not the concept sequence did not lead to a reduction of switch costs. A reduction of switch costs based on a pre-knowledge of the language sequence was only observed when the concept sequence was known as well (Experiment 1). However, it was also found that switch costs and mixing costs remained in predictable sequences, even when the upcoming language and concept were known (Declerck et al., 2013). These results indicate that even as proactive language control may be used, it cannot resolve competition completely prior to using the other language. Overall, it appears that proactive language control can be used to reduce the amount of between-language competition, though there are some mixed results as well (e.g., Philipp et al., 2007). Yet, while experiments suggest a general role for proactive language control, its scope and limitations remain to be further clarified. In this context, one underexplored boundary condition remains whether people can implement preparatory processes on the basis of statistical regularities (i.e., sequence learning). This is an important question to consider for at least two reasons: First, it allows us to test if proactive language control can indeed be implemented over an extended period of time based on a distributed signal (i.e., statistical regularities). Second, predictable sequences may in fact be one way in which language switching is monitored by bilinguals: For example, language switches are less likely to happen within a phrase (which could be construed as a type of a predictive sequence); here, we can test if people can use such information to reduce cross-lingual competition.

## The current study

The current study investigated in three experiments the role of proactive language control in non-voluntary language switching. More specifically, we asked whether participants can use information from predictable sequences to implement preparatory processes to reduce performance costs as the result of language switches following external constraints. To do so, we used a novel approach of manipulating predictability that is borrowed from research on implicit motor sequence learning (e.g., Schwarb & Schumacher, 2012, for a review) and that has currently only been implemented in the context of task switching (Koch, 2005) but not yet in language switching.

In task switching, similarly to language switching, participants are required to switch between two different task sets in turn (see Koch, Poljac, Müller, & Kiesel, 2018, for a recent review). To investigate preparatory processes, Koch (2005) had participants switch between two binary classification tasks (T1 and T2) in a simple, predictable, alternating sequence (T1, T1, T2, T2, T1, T1, etc.). In addition, participants completed one block in a random sequence, which was followed by another predictable sequence block. Performance for predictable blocks was calculated by averaging dependent variables (i.e., RTs and error rate) in the penultimate and ultimate blocks, in which the predictable sequence was implemented (i.e., the predictable blocks right before and after a random one). This approach was taken to account for possible confounding effects in the predictable sequence blocks (e.g., practice effects such as general familiarity with the stimuli; fatigue). One advantage of this approach (vs. for example implementing predictable and random blocks in alternation; c.f., Declerck et al., 2015) is that the effect of predictability can build across blocks—as would be expected from a sequence learning perspective—while also controlling for the effects of practice. This is also why this design requires the random block to always be presented in the penultimate block position. In contrast, shorter implementations of a predictable sequence may not be sufficient to observe the impact of the sequence (Koch, 2005). When comparing performance (speed and accuracy) across the predictable and random sequences, participants were found to benefit from sequential task predictability. That is, participants’ reaction times were shorter and error rates were lower when they knew in what sequence tasks had to be performed than when they did not (random sequence). Moreover, predictable sequences conveyed a benefit to repetition and switch trials equally, suggesting that predictability was not exploited to specifically prepare for task switches (Koch, 2005).

In the present study, we implemented this methodology in language switching. Participants had to name pictures in two languages. This was done in the same simple double alternation, predictable sequence pattern (L1, L1, L2, L2, L1, L1, etc.). Subsequently, they completed one test block where the predictable sequence was no longer implemented, followed by a final predictable language block. The predictability benefit is calculated by subtracting the average performance on the penultimate and final predictable sequence blocks from the random sequence block (i.e., negative transfer block). This approach then allows for the direct performance comparison per language transition (repetition, switch) and language (L1, L2) in predictable and non-predictable sequences as well as possible interactions.

In three experiments, we investigated whether and how predictability of sequences would benefit language switching. We predicted to find a predictability advantage in language switching with repetition and switch trials benefitting equally, similarly to what had been reported in task switching (Koch, 2005). Moreover, we hypothesized that performance in L2 naming trials would be quicker than L1 naming trials (due to L1 slowing), consistent with previous results (e.g., Costa & Santesteban, 2004). Such finding would be another indication of proactive language control, although we cannot specifically examine L1 slowing in terms of asymmetric language mixing costs as the experiments did not include pure language blocks. To summarize, the three experiments allowed us to address to what extent language switching benefits from predictable sequences.

To foreshadow, contrary to our expectations, we found no evidence for a general predictability benefit or a switch-specific one in Experiment 1. In contrast, there was a trend for better performance when no predictable sequence was implemented. To confirm these surprising results, we conducted Experiment 2 as a conceptual and slightly improved broad replication of Experiment 1 using an increased sample size. In Experiment 3, we significantly reduced the number of concepts participants had to name; here, we found a considerable general predictability benefit that was limited to repetition trials. The results of the three experiments are discussed together as part of the General Discussion.

## Experiment 1

### Method

#### Participants

24 students with normal or corrected-to-normal vision from RWTH Aachen University participated for course credit. All had received at least seven years of English education and were native speakers of German. One participant had to be excluded from analysis because of a coding error, leaving 23 participants in the final sample. Proficiency in German and English was measured with the Lexical Test for Advanced Learners of English (LexTALE; Lemhöfer & Broersma, 2012). Their average score for German (L1) was 87.10 and for English (L2) was 70.74. Participants were consented according to an internal ethics procedure.

#### Stimuli

36 pictures of well-known semantic concepts were selected; an overview of all stimuli used can be found in Appendix A1 (Table A1). Words were either monosyllabic or disyllabic non-cognates, and they were matched in length (M_L1_ = 5.06^1^ ± 1.31; M_L2_ = 4.58 ± 1.13, *t*(35) = 1.84, *p* = 0.074). They all had a Levenshtein distance of at least 3 (M = 4.83 ± 1.23) to limit competition based on orthographic overlap. Two translation-equivalent stimuli overlapped at onset (BRUSH/BUERSTE and TABLE/TISCH). Picture representations were selected black and white images from different databases (Bates et al., 2003; Severens et al., 2005; Snodgrass & Vanderwart, 1980). Naming language was cued with either a British or a German flag.

#### Procedure

The experiment consisted of eight blocks with 72 trials each, resulting in 576 trials. In addition, each block started with a random warm-up trial, so that the overall number of experimental trials was 584. Additionally, there was a practice block that consisted of 12 trials, which did not include the predictable sequence.

Language presentation through Blocks 1–6 and Block 8 were predictable, where there were always two naming trials of each language (e.g., L1, L1, L2, L2, L1, L1, etc.). Each picture was named twice each block, once in German and once in English. As a result of the predictable sequence, the number of repetition trials (where the language of the current and previous trial matched) and switch trials (where the language of the current and previous trial did not match) was equal within predictable sequence blocks. In addition, the same picture could not be named twice in the same row. For the random block, number of repetition and switch trials was kept roughly equal as well with a number of constraints: Trials with the same language could only repeat three times in a row; as before, the same picture could not be named twice in a row.

Before participation, all participants completed a demographics questionnaire. In addition, they received a worksheet with all pictures used in the experiment. Due to experimenter error, only 32 of 36 pictures were included on the worksheet.^2^ In the rare case that participants named a picture with a synonym (e.g., BUNNY instead of RABBIT) or were not able to name a picture, they were given the correct word to name the picture.

Participants were instructed to name pictures either in English or German and to not use filler words (e.g., “um”). They were instructed that pictures would be presented with a cue (German flag or British flag) to indicate the language in which naming had to occur. In addition, participants were instructed that cues would be presented in the previously described fixed language sequence. In the beginning of Block 7, participants were informed that the language sequence was no longer fixed but random instead. Before Block 8, they were instructed that the predictable language sequence was reinstated. The experiment was programmed in PsychoPy (Peirce et al., 2019). Stimuli were presented on a computer screen with a resolution of 1680 x 1050 pixels on a grey background.

Each trial started with the presentation of four identical language cues (185.20 x 185.20 pixels each) left, right, above and under the eventual position of to-be-named stimulus (left and right flags were offset from center by 566.93 pixels; top and bottom flags were offset by 377.95 pixels). All four cues were present on each trial. After 100 ms, the picture was presented in the center of the screen (370.39 pixels × 370.39 pixels) while the cues remained visible (i.e., the picture was also presented in the center of the cues); it remained there for 3500 ms or until a response was made and registered by a voice key (microphone: Basetech BR DM 20). There were 900 ms between a participant’s response was detected and the start of the new trial (i.e., the presentation of the next cue). An experimenter always stayed in the room to code participants’ vocal responses in real-time and to adjust the microphone if necessary. Overall, each participant’s session took between 40 and 60 minutes.

#### Design

The goal of Experiment 1 was to investigate how language sequence predictability impacted participants’ ability to switch between two languages. To analyze practice effects across the experiment (sequence learning), there were three independent variables: language (L1 or L2), language transition (repetition or switch trial) and block (1–6). To analyze the effect of predictability, the three independent variables were language (L1 or L2), predictability (predictable or random sequence), and language transition (repetition or switch trial). Scores for the predictable sequence blocks were calculated by taking the average of Blocks 6 and 8 (penultimate and final predictable blocks) by condition. The predictability benefit was calculated by subtracting the average for predictable sequence blocks from random block performance. The dependent variables were always reaction time (RT) and error rates per condition.

### Results

Analyses were implemented in R (Version 4.0.2; R Core Team, 2020). The following trials were excluded from analysis with RT as a dependent measure: practice trials, error trials, one trial after each error trial, the first trial (warm-up trial) of each block and all trials with RTs below 100 ms. To identify additional outliers, RTs were z-transformed for each participant separately; if a trial had a z-value of +3/–3, it was excluded as an outlier. For RT analyses, this procedure resulted in total in the discarding of 15.1% of all trials. The same exclusion criteria were used for analyses with error rate as a dependent variable, with the exception that only error trials were excluded that followed an error (overall 13.2% of trials discarded). Analyses were conducted on RT and error values with pre-planned ANOVAs.

#### Practice effects

**Figure 1:**
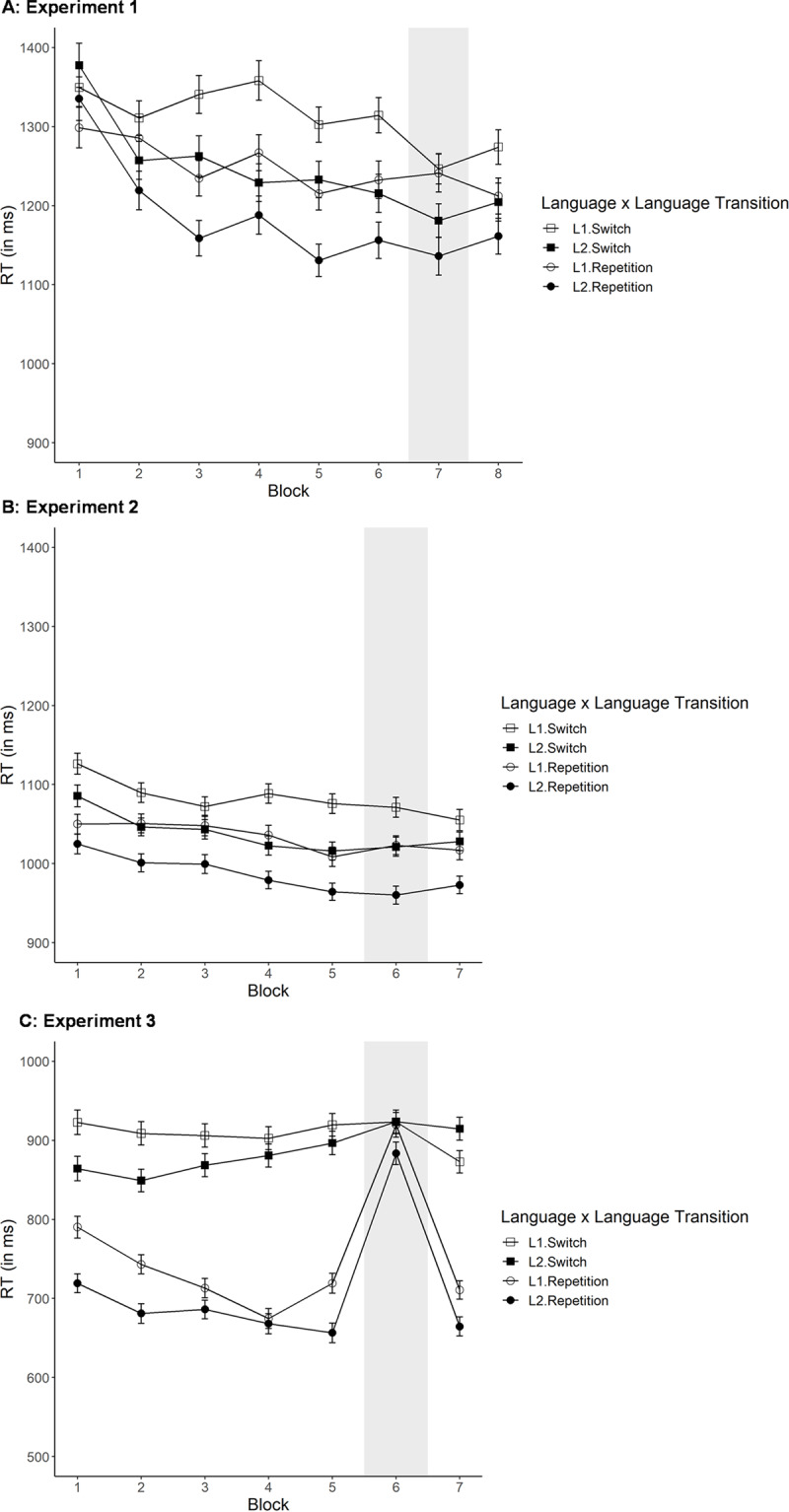
RT (in ms) across blocks for languages (L1, L2) and language transition (switch, repetition) in Experiment 1 (Panel **A**), Experiment 2 (Panel **B**) and Experiment 3 (Panel **C**). Shaded area indicates random sequence block. Error bars indicate one +/– one standard error. Please note that different y-axis values were selected for Experiment 3, as RTs were much shorter than in previous experiments.

RT data can be seen in Panel A of ***Figure 1***: In general, RTs appear to decrease over the course of the experiment. RT data were analyzed with a 2 × 2 × 6 within-subject, repeated ANOVA (language × language transition × block). We found a significant effect of block (*F*(2.58, 56.82) = 7.09, *p*(Greenhouse–Geisser corrected [GG]) < 0.001, generalized *η*^2^ = 0.0368) as a result of a general 110 ms reduction in RT across the experiment consistent with a practice effect. Moreover, there was a significant effect of language (*F*(1, 22) = 24.00, *p* < 0.001, generalized *η*^2^ = 0.01897), where participants’ naming was slower in their L1 than their L2 (M_L1_ = 1291 ms; M_L2_ = 1227 ms), and a significant effect of language transition with responses being 69 ms slower in switch than repetition trials (M_repetition_ = 1226 ms; M_switch_ = 1295 ms; *F*(1, 22) = 44.73, *p* < 0.001, generalized *η*^2^ = 0.0227). There was also a significant interaction between language and block (*F*(5, 110) = 5.18, *p* < 0.001, generalized *η*^2^ = 0.0098). To investigate this interaction, we split the data by block and repeated the analysis separately (applying the Bonferroni correction, α = 0.008). Here, we found no significant effect of language for Blocks 1–2 (F = Block 1: *p* = 0.272; Block 2: *p* = 0.038), whereas it reached significance for Blocks 3–6 (*p*s ≤ 0.001). Thus, the interaction was the result of comparable RTs for L1 and L2 in Blocks 1–2; in contrast, for subsequent blocks, performance in L2 was always quicker than performance in L1 (see also ***Figure 1A***). No other interaction reached significance (all *F*s < 2).

**Figure 2:**
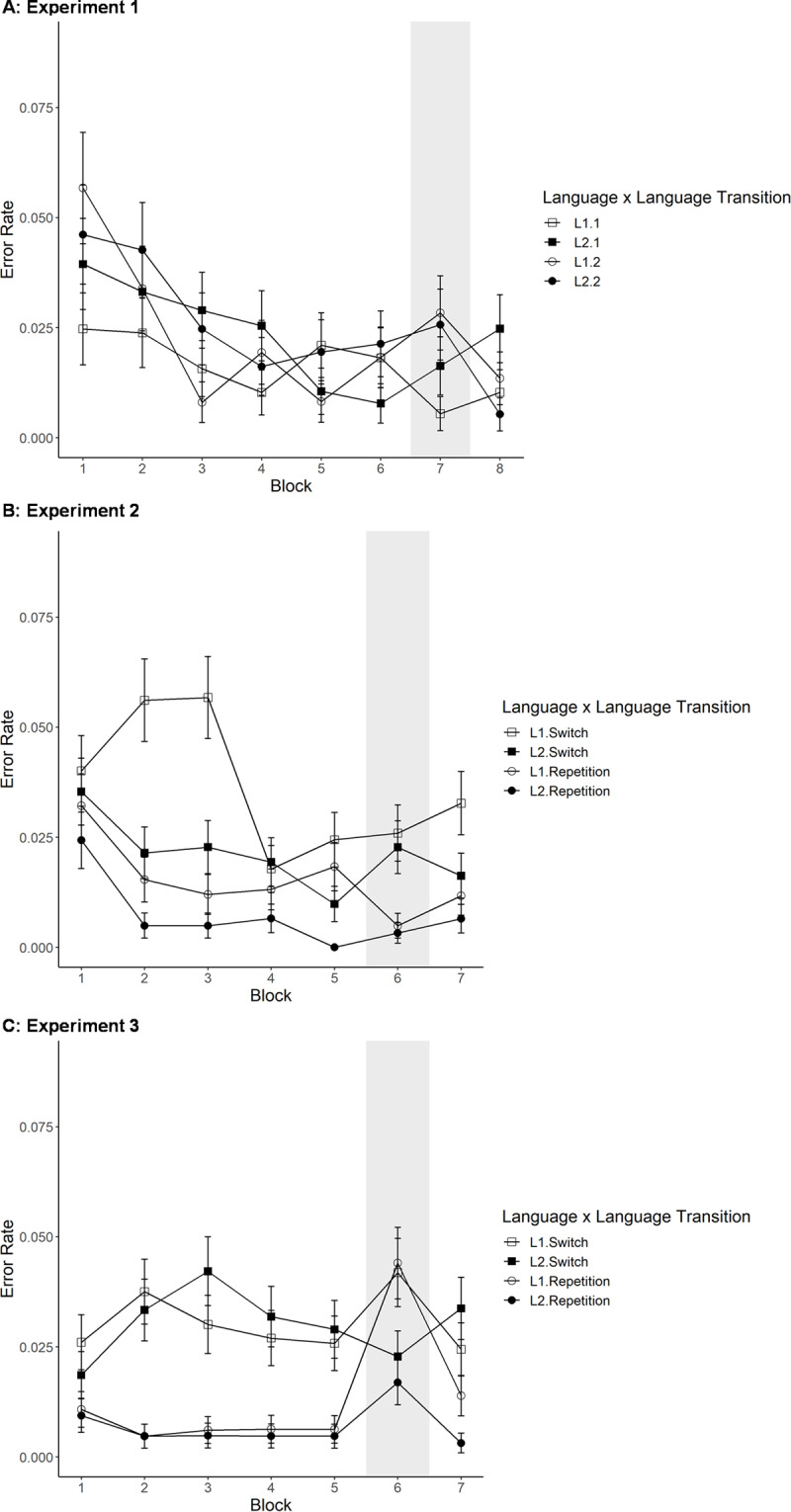
Error rate across blocks for languages (L1, L2) and language transition (switch, repetition) in Experiment 1 (Panel **A**), Experiment 2 (Panel **B**) and Experiment 3 (Panel **C**) Error rates are reported as averages across trials, where each error was coded as 1 and each accurate trial as 0. Shaded area indicates random sequence block. Error bars indicate one +/– one standard error.

Using the same type of analysis for error rate (see Panel A of ***Figure 2***), we found a significant effect of block in the same direction as before (*F*(2.73, 60.10) = 6.92, *p*(GG) < 0.001, generalized *η*^2^ = 0.0636). No other main effects or interactions reached significance (all *F*s < 2), likely due to the low overall number of errors (M = 0.022).

#### Predictability effect

**Figure 3:**
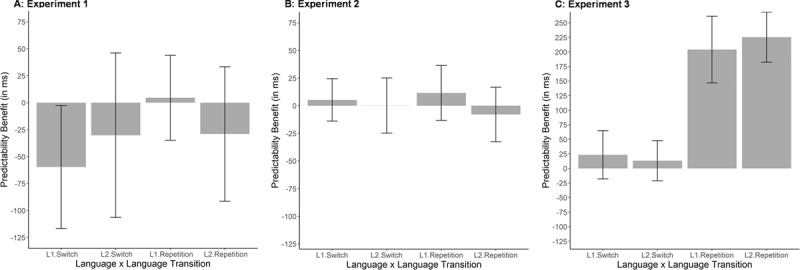
Predictability benefit (performance on random block minus performance on predictable sequence blocks) in Experiment 1 (Panel **A**), Experiment 2 (Panel **B**) and Experiment 3 (Panel **C**) for RT data. Performance for predictable sequence blocks was calculated by taking the average performance of the penultimate and final predictable blocks. Please note that different y-axis values were selected for Experiment 3, as the predictability benefit was much more pronounced than in previous experiments. Error bars across conditions indicate 95% confidence intervals.

Based on visual inspection of ***Figure 1A***, there is no clear predictability benefit (comparison of Block 7 with average of Blocks 6 and 8). We conducted a within-subject, repeated 2 × 2 × 2 ANOVA (language × language transition × predictability). To do so, we averaged RTs for the last two predictable blocks (Blocks 6 and 8) to compare to performance in Block 7, the random block. We found a significant effect of language (*F*(1, 22) = 23.65, *p* < 0.001, generalized *η*^2^ = 0.0341), where naming in L1 was slower than L2 naming (M_L1_ = 1247 ms; M_L2_ = 1170 ms), and a significant effect of language transition with shorter RTs for repetition than switch trials (switch costs = 47 ms; M_repetition_ = 1185 ms; M_switch_ = 1232 ms; *F*(1, 22) = 10.86, *p* = 0.003, generalized *η*^2^ = 0.0125). Most importantly, there was no significant effect of overall predictability (*F*(1, 22) = 3.23, *p* = 0.086, generalized *η*^2^ = 0.0048), though it was marginal; in fact, there was an unexpected 29 ms trend for shorter (rather than longer) RTs in the random block than in predictable sequence blocks (M_random_ = 1194 ms; M_predictable_ = 1223 ms). None of the interactions reached significance (all *F*s < 2). The results from these analyses are represented in Panel A of ***Figure 3***.

The same analyses were repeated with error rate as the dependent variable. Here, no main effect was significant but there was a non-significant trend for a higher error rate in switch than repetition trials (*F*(1, 22) = 3.04, *p* = 0.095, generalized *η*^2^ = 0.015). In addition, the interaction of predictability and language transition was significant (*F*(1, 22) = 4.35, *p* = 0.049, generalized *η*^2^ = 0.014). None of the other main effects or interactions reached significance (*F*s < 2). To follow-up the significant interaction of predictability and language transition, we split the data by predictability and repeated the analysis (without predictability as a factor). We found that in the random block, the effect of language transition was significant in the expected direction (*F*(1, 22) = 7.27, *p* = 0.013, generalized *η*^2^ = 0.0453), while this was not the case in the predictable block (i.e., absence of error switch costs; *F* < 1). All means per condition are summarized in Appendix B.

#### Additional analyses

Given these unexpected results, we decided to calculate Bayes Factors to estimate the strength of the evidence. As before, these analyses were implemented in R (Version 4.0.2; R Core Team, 2020). To minimize the number of model comparisons, we calculated Bayes Factor for the model without predictability and all its interactions to the fully saturated one and then compared them by taking their ratio. By doing so, we could ask to what extent adding predictability as well as its interaction helped accounting for the data. For RT analyses, this revealed that the model including predictability and its interactions as independent variable was preferred by a factor of 0.004 ± 9.73%. Similarly, for error rate analyses, this showed that the model that included predictability and its interaction was preferred only by a factor of 0.0211± 25.77%. This is consistent with strong evidence for the null hypothesis that predictability did not impact participants’ performance, though there appears to be quite a large error estimate in both analyses.

Overall, Experiment 1 showed no predictability benefit, but, if at all, rather a trend towards a predictability disadvantage. Given these surprising results, Experiment 2 was conducted as a broad conceptual replication with only few minor methodological improvements and an increased sample size.

## Experiment 2

### Method

#### Participants

35 students with normal or corrected-to-normal vision from RWTH Aachen University participated for course credit. This sample size was selected to detect effect sizes of 0.5 at power of 0.8. As before, participants’ L1 was always German and their L2 was English. Average LexTALE scores for German (L1) were 87.26 and for English were 71.14 (L2). Participants were consented consistent with an internal ethics procedure.

#### Stimuli

Stimulus pictures were selected to represent 20 semantic concepts (Appendix A1 Table A2); they were chosen to cover a range of semantic areas to avoid a high number of closely related concepts. Semantic concepts were selected to match onto German and English monosyllabic, high-frequency words. In addition, translation-equivalents were matched in length (M_L1_ = 4.1 ± 0.64; M_L2_ = 4.2 ± 0.83; *t*(19) = -0.57, *p* = 0.577). All translation-equivalents (e.g., HORSE and PFERD) were non-cognates and had a Levenshtein distance of at least 2 (M = 3.9 ± 0.91) to guarantee that competition between items was not solely based on orthographic overlap. Only two items overlapped at the beginning (BOOK/BUCH and GOOSE/GANS). Colored pictures of semantic concepts were selected from the database MultiPic (Duñabeitia et al., 2018).

#### Procedure

The experiment consisted of seven blocks.^3^ Each block consisted of four runs of 20 trials (i.e., each run included each of the 20 pictures once, but there was no pause between them), thus resulting in an overall number of 80 trials per block and 560 trials in total. In six of the seven blocks, naming had to occur in the same fixed language sequence where two subsequent trials were always of the same language, resulting in an equal number of switch and repetition trials. Participants completed five blocks in which the fixed language sequence was implemented. This followed by one block with a random sequence (Block 6) and a final block where the language sequence was reinstated.

For half of the participants, the first cue of the first run of Block 1 was L1, whereas for the other half of participants the first cue of Block 1 was L2. Subsequent blocks took turns with which language cue they started (e.g., if Block 1 started with a L1 naming event, Block 2 started with a L2 naming event) with the exception of Block 6. In the random block, the sequence of language cues was randomized within each run separately for each participant with the constraint that trials with the same language cues could not appear more than three times in a row within a run and that the number of repetition and switch trials remained roughly even. These constraints were used to prevent long runs of repetition trials only. In contrast to Experiment 1, there was no random warm-up trial; thus, we always excluded the first two trials of each block and each run—that is, each time the sequence was restarted. Within a block, each semantic concept was named four times, twice in each language distributed across the four runs. The order of semantic concepts within a run was random. There were twenty practice trials (one run) with the fixed language sequence. The first cue of the practice trials matched the first cue of Block 1.

As before, participants were presented with the twenty pictures that were used in the experiment on a work sheet that they had to name in their L1 and L2. Before the start of experimental trials, participants completed the twenty practice trials; they received the same instructions as in Experiment 1. The experiment was implemented in SR Research’s Experiment Builder (SR Research Experiment Builder, 2011).

Each trial started with a centrally located cross that was flashed on a computer screen (1920 × 1080 pixels) for 50 ms. This was followed by blank screen for 50 ms. Subsequently, the four language cues (four German or British flags; 100 × 80 pixels each) were presented for 200 ms before the picture (300 × 300 pixels) came on; as before, the cues remained visible during picture presentation. The picture was centrally located, whereas the cues were always located 50 pixels offset to the four sides of the picture. A trial ended when a participant’s response was recorded by a voice key (microphone: Sennheiser e835s). After the voice response was detected, there was a delay of 1400 ms before a new trial was started to allow for task preparation. The delay was increased in comparison to Experiment 1 to allow for more preparation time between trials. If no response was detected within 3000 ms, participants were given a warning message before the next trial was started. An experimenter stayed in the room throughout the experiment to code the accuracy of vocal responses of each trial.

#### Design

Experiment 2 was a conceptual replication of Experiment 1 with an increased sample size. As for Experiment 1, the independent variables were language (L1 or L2), language transition (repetition or switch trial) and block (1–5) for the practice effect analyses. Similarly, for the predictability effect analyses, language (L1 or L2), language transition (repetition or switch trial) and predictability (fixed or random sequence) were the independent variables. The dependent variables were always reaction time (RT) and error rates per condition.

### Results

As before, analyses were implemented in R (Version 4.0.2; R Core Team, 2020) and the same exclusion criteria were used as in Experiment 1, with the exception that the first two trials of each sequence were excluded. This led to the discarding of 18.09% of the RT data and 16.38% of the error data.

#### Practice effects

Practice effects were analyzed for Blocks 1–5, in which the predictable sequence was implemented. RT data (see Panel B of ***Figure 1***) were entered into the same within-subject, repeated measures ANOVA as before with block (1–5), language (L1, L2) and language transition (repetition, switch) as independent variables.

We found a significant effect of block (*F*(2.93, 99.48) = 7.42, *p*(GG) < 0.001, generalized *η*^2^ = 0.0143), consistent with practice effects (shorter RTs for later blocks; RT difference between mean of Block 1 and mean of Block 5 = 56 ms). There was also a significant effect of language (*F*(1, 34) = 16.80, *p* < 0.001, generalized *η*^2^ = 0.0210), where RTs were longer for L1 than L2 naming trials (L1 slowing; M_L1_ = 1065 ms vs. M_L2_ = 1018 ms). As expected, RTs were also significantly shorter in repetition trials than in switch trials (M_repetition_ = 1016 ms; M_switch_ = 1066 ms; *F*(1, 34) = 119.66, *p* < 0.001, generalized *η*^2^ = 0.0246). Finally, the interaction between language transition and block reached significance (*F*(4, 136) = 2.52, *p* = 0.044, generalized *η*^2^ = 0.0015). None of the other interactions reached significance (all *F*s < 1.5). To investigate the interaction between language transition and block, we split the data by block and repeated it. We found that the effect of language transition remained significant in all blocks (*p* < 0.001). Inspecting the switch costs more closely (see Table B3 in the appendix for means of all conditions), it can be seen that switch costs differed the most between Block 1 and Block 3—there was a reduction of switch costs between Block 1 and 3; however, switch costs then again increased between Blocks 4 and 5 (similar to a U-shaped pattern). This pattern does not appear to be systematic (nor was it observed in Experiment 1), and is therefore not further analyzed here.

When repeating these analyses with the error rates, we found exactly the same pattern of significant main effects (block: *F*(3.08, 104.70) = 9.00, *p*(GG) < 0.001, generalized *η*^2^ = 0.0400; language: *F*(1, 34) = 22.90, *p* < 0.001, generalized *η*^2^ = 0.0354; language transition: *F*(1, 34) = 33.57, *p* < 0.001, generalized *η*^2^ = 0.0548) in the same directions as before. In addition, all interactions with block reached significance (language × block: *F*(4, 136) = 2.57, *p* = 0.041, generalized *η*^2^ = 0.0130; language transition × block: *F*(3.08, 104.65) = 3.38, *p*(GG) = 0.020, generalized *η*^2^ = 0.0209; language × language transition × block: *F*(4, 136) = 2.61, *p* = 0.039, generalized *η*^2^ = 0.0117). Inspecting the data visually (Panel B of ***Figure 2***), it appears that these effects were likely driven by the high error rate that was isolated to L1 switch trials in the first half of the experiment. To investigate this statistically, we again split the data by block and repeated the analysis (without block as a factor; applying the Bonferroni correction). The effect of language was significant in all blocks (Block 2: *F*(1, 34) = 12.24, *p* = 0.001, generalized *η*^2^ = 0.0886; Block 3: *F*(1, 34) = 10.32, *p* = 0.003, generalized *η*^2^ = 0.0770; Block 5: *F*(1, 34) = 11.93, *p* = 0.001, generalized *η*^2^ = 0.0687) but Block 1 and 4 (*F*s < 1.2) with more errors for the L1 than L2. The effect of language transition only reached significance in Blocks 2 (*F*(1, 34) = 21.65, *p* < 0.001, generalized *η*^2^ = 0.1305) and 3 (*F*(1, 34) = 21.67, *p* < 0.001, generalized *η*^2^ = 0.1587). The interaction of language and trial transition was never significant (but was marginal in Block 3 after applying the Bonferroni correction: *F*(1, 34) = 6.33, *p* = 0.017, generalized *η*^2^ = 0.0353; all other *F*s < 3.6). Together, it seems hard to find a clear explanation for this specific pattern of practice effects in the error rates, possibly because error rate was generally low, so that small variations can result in interaction patterns.

#### Predictability effect

To calculate the effect of predictability, we averaged RTs and error rates for predictable sequence Blocks 5 and 7 and compared them to performance in random sequence Block 6. To do so, we used the same ANOVA structure as in Experiment 1, investigating the effects of language, language transition and predictability as well as their interactions. For RT data, only the main effects of language (*F*(1, 34) = 22.26, *p* < 0.001, generalized *η*^2^ = 0.0222) and language transition (*F*(1, 34) = 82.91, *p* < 0.001, generalized *η*^2^ = 0.0272) reached significance, indicating shorter RTs in L2 (M = 995 ms) than in L1 (M = 1044 ms) as well as in repetitions (M = 992 ms) than in switches (M = 1047). Importantly, the main effect of predictability was not significant (*F* < 1) nor were any of the interactions (*F* < 1.5). The predictability results from the RT analyses of Experiments 2 (Panel B) are presented in ***Figure 3*** and again highlight the absence of a predictability benefit across the first two experiments.

When analyzing error rates, the main effect of language (*F*(1, 34) = 8.58, *p* = 0.006, generalized *η*^2^ = 0.0242) and language transition (*F*(1, 34) = 15.90, *p* < 0.001, generalized *η*^2^ = 0.0867) reached significance with effects in the same direction as before. The main effect of predictability did not reach significance (*F* < 0.5). However, there was a non-significant trend for an interaction of language and predictability (*F*(1, 34) = 3.73, *p* = 0.062, generalized *η*^2^ = 0.0109): For L1, there was a trend towards more errors in predictable than in random blocks (*F*(1, 34) = 3.26, *p* = 0.080, generalized *η*^2^ = 0.0129), whereas that was not the case for L2 naming trials (*F*(1, 34) = 1.19, *p* = 0.282, generalized *η*^2^ = 0.0089). Hence, if at all, we found some “costs” of predictability in the error rates in Experiment 2, which is consistent with the finding of Experiment 1 that, if at all, there was some predictability cost for RT trials. (None of the other main effects or interactions reached significance in the error rates, *F* < 2.5.) All means per condition are given in Appendix B.

#### Additional analyses

As for Experiment 1, we calculated Bayes Factor for the model that included predictability and its interactions to the one without and then calculated the ratio. For RTs, this revealed that the model with predictability and its interactions was preferred by a factor of < 0.001 ± 5.78%. For error rates, the model with predictability and its interactions again was not preferred (factor = 0.010 ± 11.47%). Together, against our predictions, these results are consistent with strong evidence for the null hypothesis, i.e. that the influence of predictability was non-existent.

Overall, consistent with Experiment 1, Experiment 2 again showed no predictability benefit. Participants were not able to use the predictable sequences to facilitate performance and/or language switching. In Experiment 3, we significantly decreased the number of semantic concepts that participants had to switch between to two; thus, we investigated whether a predictability effect could be observed if there was less ambiguity about the concept that needed to be named on a subsequent trial.

## Experiment 3

### Method

#### Participants

37 German-English unbalanced bilinguals with normal or corrected-to-normal vision from RWTH Aachen University participated for course credit. They again had received at least seven years of English education and were native speakers of German. Their LexTALE score for German was 89.14 and for English was 73.94, and they were consented according to an internal ethics procedure. Two participants had to be excluded because they had participated in Experiment 2, which had only been identified after data collection.

#### Stimuli

Two pictures of well-known semantic concepts were selected: LEG/BEIN and KLEID/DRESS. Translation-equivalents were non-cognates, they were similar in length^4^ (M_L1_ = 4.50 ± 0.71; M_L2_ = 4.00 ± 1.41) and had a Levenshtein distance of at least 3 (M = 3.5 ± 0.71). Pictures were selected from the same database as in Experiment 2 (MultiPic; Duñabeitia et al., 2018).

#### Procedure

As in Experiment 2, the experiment consisted of seven blocks with 80 trials each and 560 trials in total. In six of the seven blocks (Blocks 1–5 and Block 7), naming again occurred in the same fixed language sequence as before. In Block 6, naming occurred in a random sequence.

The first language cue of each block was counterbalanced across participants, so that for half the participants it was L1 and for the other half it was L2, with subsequent blocks taking turns with which language cue they started. Within a run of four trials, each semantic concept was named once in the L1 and once in the L2. The order of semantic concepts within a run was random. Order across runs was pseudo-random to prevent complete repetitions (both picture and response repetitions); however, in contrast to previous experiments, it was possible that the same picture was repeated across runs (this was unavoidable given the small number of concepts). As before, in the random block, trials with the same language cues could not appear more than three times in a row with a roughly even number of switches and repetitions. As for the predictable blocks, picture repetitions were possible, while complete repetitions were not. Within a block, each semantic concept was named forty times, twenty times in each language. Again, there was a practice block of twenty trials with a fixed language sequence that participants completed before the experimental trials started. Participants again had to fill out a worksheet with pictures of the used concepts and name them in both their L1 and L2. They received the same instructions as before. The experiment was implemented in SR Research’s Experiment Builder (SR Research Experiment Builder, 2011).

The trial procedure was identical to Experiment 2. The only exception was that the experimenter did not stay in the room throughout the experiment for all participants. As an adaptation to the COVID-19 pandemic, for approximately half of the participants, the experimenter was positioned outside the lab space during testing. They monitored the participant’s vocal responses via Bluetooth headphones and coded them for accuracy on the go as before.

#### Design

The design was identical to Experiment 2 with the only significant exception being the number of semantic concepts that had to be named. For the practice effect analyses, the independent variables were (L1 or L2), language transition (repetition or switch trial) and block (1–5). For the predictability effect analyses, they were language (L1 or L2), language transition (repetition or switch trial) and predictability (fixed or random sequence). The dependent variables were always reaction time (RT) and error rates per condition.

### Results

Analyses were implemented in R (Version 4.0.2; R Core Team, 2020) and the same exclusion criterion were used as in Experiment 1. This led to the discarding of 12.34% of the RT data and 10.51% of the error data.

#### Practice effects

Practice effects were analyzed for predictable Blocks 1–5 in the same within-subject, repeated measures ANOVA as for Experiment 2. RT data are presented in Panel C of ***Figure 1***. We found no significant effect of block (*F*(2.53, 86.02) = 2.50, *p*(GG) = 0.075, generalized *η*^2^ = 0.0085), but significant main effects of language (*F*(1, 34) = 34.75, *p* < 0.001, generalized *η*^2^ = 0.0196) and language transition (*F*(1, 34) = 142.10, *p* < 0.001, generalized *η*^2^ = 0.2712): RTs were longer when naming pictures in L1 than L2 trials (L1 slowing; M_L1_ = 820 ms vs. M_L2_ = 778 ms) and for switch than repetition trials (M_repetition_ = 705 ms; M_switch_ = 892 ms). In addition, the interaction of language transition and block reached significance as well (*F*(2.86, 97.07) = 7.65, *p*(GG) < 0.001, generalized *η*^2^ = 0.0109). All other interactions did not reach significance (*F*s < 2.3). Visual inspection of ***Figure 1C*** strongly suggests that the interaction of language transition and block was driven by differences in performance in switch and repetition trials.^5^ Thus, in contrast to previous experiments, we decided to split the data by language transition to investigate its interaction with block, thus asking whether potential practice effects were limited to one type of transition (applying the Bonferroni correction, α = 0.025). It was found that block only reached significance for repetition (*F*(2.69, 91.62) = 8.10, *p*(GG) < 0.001, generalized *η*^2^ = 0.0399) but not switch (*F* < 1) trials, indicating that improvements over time could only be observed for repetitions.

We replicated these analyses with error rate as the dependent measure. It should be noted that overall error rate was very low in Experiment 3 (M = 0.0184). The only effect that reached significance was the one of language transition (*F*(1, 34) = 39.35, *p* < 0.001, generalized *η*^2^ = 0.1125): Participants made more errors for switch than repetition trials (M_repetition_ = 0.006; M_switch_ = 0.030). In addition, the interaction of language transition and block was right at the border to significance (*F*(4, 136) = 2.43, *p* = 0.0503, generalized *η*^2^ = 0.0102; all other *F*s < 1). Consistent with the RT analyses, we split the data by language transition to further investigate this interaction. Whereas there was a significant effect of block for repetition trials (indicating improvements over time; *F*(2.59, 87.91) = 7.88, *p*(GG) < 0.001, generalized *η*^2^ = 0.0380), this was not the case for switch trials (*F* < 1).

#### Predictability effect

We again calculated the predictability effect by averaging RTs and error rates for predictable sequence Blocks 5 and 7 and compared them to performance in random sequence Block 6. To do so, we used the same ANOVA structure as before.

For RT analyses, we found a significant effect of language transition (*F*(1, 34) = 89.77, *p* < 0.001, generalized *η*^2^ = 0.1090) but not language (*F*(1, 34) = 3.44, *p* = 0.072, generalized *η*^2^ = 0.0039). Importantly, the effect of predictability reached significance (*F*(1, 34) = 54.87, *p* < 0.001, generalized *η*^2^ = 0.1123): RTs were overall shorter in the predictable (M = 792 ms) than in the random block(s) (M = 909 ms). In addition, the interaction between language transition and language (*F*(1, 34) = 10.86, *p* = 0.002, generalized *η*^2^ = 0.0056) as well as the one between language transition and predictability (*F*(1, 34) = 90.16, *p* < 0.001, generalized *η*^2^ = 0.0824) reached significance. To investigate these interactions, we again split the data by language transition (consistent with the practice effect analyses of Experiment 3). Here, we found that the effects of language (L1 slowing; *F*(1, 34) = 10.38, *p* = 0.003, generalized *η*^2^ = 0.0191) and predictability (*F*(1, 34) = 95.87, *p* < 0.001, generalized *η*^2^ = 0.3054) were significant for repetitions; at the same time, this was not the case for switch trials (*F*s < 1.5). As a complimentary post-hoc analysis, we also repeated this analysis but split the data by predictability instead. Here, we found no significant effects for the random block (all *F*s < 1.5); however, for the predictable blocks, there were significant switch costs (*F*(1, 34) = 170.58, *p* < 0.001, generalized *η*^2^ = 0.3366) as well as a significant interaction of trial transition and language (*F*(1, 34) = 12.39, *p* = 0.001, generalized *η*^2^ = 0.0116).

In addition, we decided to do an additional post-hoc analysis to better understand why we did not find any predictability benefits for performance on language switch trials. Here, we hypothesized that picture repetitions across runs led to negative priming, thus additionally impeding performance after a language switch (direct picture repetitions were not allowed in Experiments 1–2 but were possible in Experiment 3). Thus, we replicated this analysis after removing all trials in which there was a direct picture repetition. Given that this exclusion and analysis was post-hoc, results should be treated as exploratory. We found the pattern of significance to be exactly the same as for the data analysis that included picture repetitions: We found a significant effect of language transition (*F*(1, 34) = 65.31, *p* < 0.001, generalized *η*^2^ = 0.0824) and predictability (*F*(1, 34) = 30.28, *p* = 0.003, generalized *η*^2^ = 0.0780) in addition to the interactions of language transition and language (*F*(1, 34) = 9.70, *p* = 0.004, generalized *η*^2^ = 0.0094) as well as predictability and language transition (*F*(1, 34) = 103.31, *p* < 0.001, generalized *η*^2^ = 0.0960). We did not further investigate the significant interactions, given the already small number of trials per cell in these exploratory analyses (N ≥ 7 for switch trials). However, these results suggest that negative priming was not the reason why language switches did not benefit from predictability.

For error rate, the main effects of language (*F*(1, 34) = 9.09, *p* = 0.005, generalized *η*^2^ = 0.0272), language transition (*F*(1, 34) = 9.39, *p* = 0.004, generalized *η*^2^ = 0.0281) and predictability (*F*(1, 34) = 10.93, *p* = 0.002, generalized *η*^2^ = 0.0413) reached significance: Participants made more errors for L1 (M = 0.031) than L2 (M = 0.019), switch (M = 0.031) than repetition (M = 0.019) trials as well as random (M = 0.032) than predictable (M = 0.018) blocks. In addition, the interactions of language and predictability (*F*(1, 34) = 9.26, *p* = 0.004, generalized *η*^2^ = 0.0268) as well as of language transition and predictability (*F*(1, 34) = 9.57, *p* = 0.004, generalized *η*^2^ = 0.0190; all other *F*s < 1.6) were significant. To further investigate the interactions, we split the data by predictability and repeated the analyses. For the random block, only the effect of language was significant (*F*(1, 34) = 12.77, *p* = 0.001, generalized *η*^2^ = 0.0710), with more errors for L1 than L2 (all other *F*s < 1). In contrast, for the predictable blocks, only the main effect of language transition was significant (more errors for switch than repetition trials; *F*(1, 34) = 22.60, *p* < 0.001, generalized *η*^2^ = 0.1488; all other *F*s < 2.9).

We again conducted the exploratory post-hoc analysis where all trials with picture repetitions were excluded. We found significant main effect of language (*F*(1, 34) = 10.16, *p* = 0.003, generalized *η*^2^ = 0.0291), language transition (*F*(1, 34) = 14.58, *p* < 0.001, generalized *η*^2^ = 0.0511) and predictability (*F*(1, 34) = 12.19, *p* = 0.001, generalized *η*^2^ = 0.0441) as before. In addition, the interaction between language and predictability again reached significance (*F*(1, 34) = 16.00, *p* < 0.001, generalized *η*^2^ = 0.0533). In contrast to the analysis that included all trials, the interaction between language transition and predictability was no longer significant (*F* < 1.5), but the three-way interaction was (*F*(1, 34) = 6.96, *p* = 0.012, generalized *η*^2^ = 0.0137). As before, all means per condition are summarized in Appendix B.

#### Additional analyses

We again calculated Bayes Factor for the model that included predictability and its interactions to the one without and then calculated the ratio. In contrast to previous experiments, the model including predictability and its interactions as independent variable was preferred by a factor of 157704201 ± 5.18%% for RT analyses and by a factor of 159638992 ± 5.37% for error rate analyses. This is consistent with extreme evidence for rejecting the null hypothesis.

In summary, Experiment 3 showed a very clear general predictability benefit for overall performance, where both RTs were shorter and errors reduced for predictable than random sequence blocks. This suggests that proactive language control can only be implemented via predictability if the number of predicted items is small. Interestingly, the benefits of predictability appear to be limited to language repetition trials, not switches. Thus, there was still no switch-specific predictability benefit.

## General Discussion

The goal of Experiments 1 to 3 was to investigate whether predictable language sequences benefitted unbalanced bilinguals’ ability to name pictures either in their L1 or L2. If yes, this would constitute evidence for proactive language control: People may be able to proactively inhibit the incorrect language or activate more the correct language if they know what language they have to use on the next trial. Surprisingly, results from Experiments 1 and 2 are not consistent with this hypothesis: There was no general nor a switch-specific predictability benefit. However, if at all, there was a non-significant trend for naming to be facilitated in the random block and not in the predictable sequence ones. Generally, our results from Experiments 1 and 2 suggest no consistent and clear benefit of sequential language predictability in language switching, and this finding is further supported by Bayes Factors, which suggested evidence in favor of the absence of a predictability benefit. When, however, the number of the to-be-named concepts was significantly reduced in Experiment 3, we did find a clear general predictability effect (i.e., better overall performance in predictable than random block(s)). RT analyses suggest that this effect was driven by better performance over the course of the experiment in repetition—but not switch—trials. Interestingly, performance in repetition trials decreased so much in the random block that we were no longer able to observe switch costs there. Together, our results indicate a predictability benefit in language switching that is very limited in scope and, importantly, not driven by changes in performance on switch trials.

Beyond our predictability results, findings were consistent with ones from other language switching studies (see for a review Declerck & Philipp, 2015): Participants showed performance consistent with L1 slowing in all three experiments, were L1 naming—despite higher L1 proficiency—was slower than for L2; this is consistent with L1 being inhibited more strongly in mixed language blocks due to its increased dominance in unbalanced bilinguals (e.g., Green, 1998). That is, our results show performance consistent with one effect that is typically attributed to proactive language control (i.e., L1 slowing), although we did not observe proactive control based on predictability. In addition, we observed switch costs as expected: In a switch trial, it is harder to activate the correct language, as it had been inhibited on the previous trial. Interestingly, we did not find any evidence for asymmetrical switch costs (i.e., more pronounced switch costs for L1 than L2) across experiments; while asymmetrical switch costs have been reported under some circumstances, recent evidence indicates that they may not be as robust as previously thought (Gade et al., 2021)—our data are consistent with that conclusion. Overall, these results suggest that implementing a predictable sequence did not fundamentally change how pictures were named.

One possible explanation for our predictability results is that knowing what language comes next increased activation of all (used) words of that language; in this scenario, a type of proactive language control could have been utilized (Declerck, 2020), but it might have had a negative or net zero effect in the predictable sequence blocks because it generally increased language competition primarily at the expense of L1. In Experiment 1, due to the use of 36 stimuli, on each switch trial in a predictable language sequence, each stimulus picture had the probability of 1/35 to occur (the probability was less later on in the sequence, but participants likely were not able to track this). Even in Experiment 2, which included a lower number of stimuli than Experiment 1, responses on each trial were 1/19 in the predictable blocks (again only in the beginning of the sequence). In contrast, in Experiment 3, there were only two possible concepts on each switch trial of the predictable sequence blocks. Moreover, on repetition trials, both the semantic concept and language could be prepared. As a result, participants may have been able to use this information to anticipate what exact concept needed to be named, even before the corresponding picture was presented.

Our design is comparable to the one used by Declerck et al. (2015): Participants had to name numbers and switch between languages in a predictable or random sequence, where either only the to-be-used language, the to-be-named concept, or both were known. Using a design in which participants knew the language but not the concept (similar to our method in Experiments 1–2 and, to some extent, Experiment 3), they found a general predictability benefit (43 ms) in Experiment 3. One possible reason why Declerck et al. (2015) showed a benefit of predictability may be that the number of responses was much smaller in their study than our first two, as only five different concepts had to be named, thus potentially making it easier to prepare a response or a small set of responses for an upcoming trial. This explanation is consistent with our substantial general predictability benefit in Experiment 3, where the number of to-be-named concepts was even smaller.

At this point, our empirical support for effects of language predictability is limited. There was no evidence for a predictability benefit if switching is required for a larger (but nevertheless constrained) set of items. Moreover, the non-significant trend for predictability costs is not reliable, as it was found for different dependent variables (RT vs. error rates) across experiments, and the exact pattern of the effect was not replicated either. The predictability benefit that we did observe (Experiment 3) appears to be driven by performance gains in repetition trials where both the language and semantic concept can be anticipated within the predictable sequence. Such a benefit would seem to be consistent with a more “local” anticipation (similarly to performance gains due to longer preparation times), rather than the longer-term anticipation that we tried to investigate here. Of course, it is possible that there is a predictability benefit for switching between larger item sets as well, but that its effect size is much smaller than what was assumed. However, the size of the effects or, more adequately, its absence (e.g., see ***Figure 3***) make us confident that there is no hidden language predictability benefit in Experiments 1 and 2. This conclusion is also supported by our Bayes Factor analyses that suggested that little was gained by adding predictability as an independent variable in our models.

One potential limitation of our manipulation is that it could have had unintended effects: More specifically, participants may have used extra cognitive resources when they actively tried to implement the predictable sequence. This could have slowed down overall processing in predictable sequence blocks. According to this account, slowing was not necessarily the result of some type of language control, but rather the consequence of a more general cognitive process. While there was a trend for longer RTs in the predictable blocks than the random one in Experiment 1 that would be consistent with the use of extra cognitive resources, there was no such pattern in Experiments 2 and 3. Evidence against this hypothesis also comes from task switching, where the same simple, predictable sequence was used to test the impact of preparatory processes on switching (Koch, 2005; see also Gotler, Meiran, & Tzelgov, 2003; Heuer, Schmidtke, & Kleinsorge, 2001; Tornay & Milán, 2001).

In addition, error rate results may be interpreted cautiously, as pre-exposure to pictures could have benefitted L2 naming more than L1 naming given that participants were unbalanced bilinguals. This could have influenced how pictures were named in the two languages. However, we are confident that the effect of pre-exposure to pictures was limited, as all stimuli were selected to be high-frequency and were known by the overwhelming majority of participants in both L1 and L2.

Moreover, some minor methodological differences between the experiments existed (especially Experiments 1 and 2), such as the number of stimuli and blocks. We see this as a strength, as it allows us to generalize our conclusions to more than one single parameter setting. This is particularly true, as there was no strong a priori reason to believe that results should have been impacted by the exact to-be-named pictures, number of blocks, etc. Nevertheless, this approach also includes some risks. For example, participants in Experiment 1 had overall higher RTs than participants in Experiment 2 (e.g., Panels A and B of ***Figure 1***). One reason for the differences we observe here may be that Experiment 1 also used two-syllable words which may require longer preparation times (e.g., Santiago, MacKay, Palma, & Rho, 2000; but see Meyer, Roelofs, & Levelt, 2003). Given these variations (and others), it is unclear why exactly differences in results emerged across Experiments 1 and 2. Nevertheless, the most important finding—the absence of a predictability benefit—is consistent across the two experiments.

Together, our experiments show that language sequence predictability did not benefit people’s ability to name pictures either quickly or correctly, unless both semantic concept and language could be anticipated. This suggests that participants could not use predictability of language sequences endogenously to employ proactive language control to reduce interference from a competing language under other conditions. Instead, what we may observe in Experiments 1 and 2 could be more adequately described as unspecific readiness for using two languages in rapid alternation, which does not differ in predictable and non-predictable blocks. Such preparation for a dual language context would be much more general than the more specific preparatory processes that were hypothesized originally. These unexpected findings stand in contrast with previous research on proactive language control: The latter suggests that while it is hard, if not impossible, to completely abolish costs from language switching, preparatory processes can reduce interference and in turn facilitate switching when longer preparation times are provided (Declerck et al., 2013, 2015; Fink & Goldrick, 2015; Mosca & Clahsen, 2016). While Costa and Santesteban (2004) also found that longer preparation times reduced switch costs, they did not find that it impacted L1 slowing—the latter finding is consistent with our findings here, indicating that preparation benefits may be limited to some circumstances only.

Further, our results may also have implications for the task switching literature. When implementing a predictable, alternating task sequence, Koch (2005) observed better performance in blocks with a predictable sequence than in a block with a random sequence. Here, participants were presented with digits one to nine (excluding five) and had to switch between deciding whether the presented number was odd/even or smaller/greater than five. That is, the number of responses was the same as in Experiment 3 (two per language/task), where we also found a general predictability benefit. Interestingly, in task switching research it is commonly found that a long preparation time reduces switch costs (e.g., Rogers & Monsell, 1995). One might speculate that preparatory effects in task switching and language switching have a different influence on performance, albeit showing a similar neurophysiological pattern in EEG studies (Lavric et al., 2019). More specifically, while preparation in task switching might lead to an activation of a small number of task-relevant stimulus-response rules, preparation in language switching using a naming task might be more abstract, as there are stimulus-response rules that are specific for each individual stimulus (and its verbal response). In this line, it can also be explained why Experiment 3 and Declerck et al. (2015) found a general preparation benefit in language switching with a small set of different stimuli. At this point, this leaves unclear whether the predictability benefit in task switching would persist if participants had to switch between a higher number of responses per task or whether any predictability benefit—in task or language switching—is ultimately subject to general cognitive limitations, independently of any other additional control processes that may be at play.

To summarize, we were able to observe behavioral patterns consistent with L1 slowing in unbalanced German-English bilinguals. Moreover, predictability benefited picture naming only when the number of to-be-produced concepts was small; performance in language switch trials was never improved. At this point, it is unclear why proactive language control may be implemented in some circumstances but not others. Future research should investigate in more detail under what circumstances people are able or unable to facilitate language switching when additional information on language order is provided via a predictable sequence. Here, we offered one further piece of evidence that endogenous proactive language control on the basis of statistical regularities in the language output, while helpful in some situations, may not benefit language switching in unbalanced bilinguals.

## Data Accessibility Statement

The data for all experiments are available at: *https://osf.io/db9ne/?view_only=66ca70209a0542de9cf7838db3502c94*.

## Additional Files

The additional files for this article can be found as follows:

10.5334/joc.219.s1 Appendix A. Overview of stimuli used.

10.5334/joc.219.s2 Appendix B. Overview of means per condition.

## Footnotes

Please note that length for German stimuli that included an umlaut (e.g., BUERSTE), the German spelling was used to calculate length (e.g., BÜRSTE).

The ones missing were TREE/BAUM, GLASSES/BRILLE, WOOD/HOLZ and WALL/MAUER.

Experiment 2 was shorter than Experiment 1 for no other reason than to reduce the overall length of the experiment.

No t-test to statistically compare length was conducted given that there were only two stimulus pairs.

To be transparent, Experiment 3 was conducted based on reviewers’ feedback significantly after the completion and analysis of Experiments 1–2. Thus, we decided to stick with our original analyses of Experiments 1–2 (where splitting by block was decided to explore the interactions).
