# Supplementary material for: Assessing Proactive Language Control: Does Predictability of Language Sequences Benefit Language Switching?
Source: J Cogn. 2022 Apr 11;5(1):27. doi: 10.5334/joc.219 (PMC9400636; doi:10.5334/joc.219)
Supplement: Appendix A. — Overview of stimuli used. [file joc-5-1-219-s1.pdf]

Appendix

A: Overview of stimuli used

Table A1: Overview of semantic concepts/translation-equivalents used in Experiment 1.

German (L1)	English (L2)
Auge	Eye
Auto	Car
Baum	Tree
Bein	Leg
Birne	Pear
Blatt	Paper
Brief	Letter
Brille	Glasses
Buerste	Brush
Fenster	Window
Guertel	Belt
Hals	Neck
Hase	Rabbit
Holz	Wood
Hund	Dog
Kind	Child
Kirsche	Cherry
Knochen	Bone
Knopf	Button
Mais	Corn
Mauer	Wall
Messer	Knife
Muell	Trash
Pferd	Horse
Puppe	Doll
Schluessel	Key
Stift	Pen
Teppich	Carpet
Tisch	Table
Tuer	Door
Uhr	Clock
Vogel	Bird
Wurst	Sausage
Zaun	Fence
Ziege	Goat
Zwiebel	Onion

Table A2: Overview of semantic concepts/translation-equivalents used in Experiment 2. Semantic concepts that were also used in Experiment 3 are marked with an asterisk.

German (L1)	English (L2)
Bein*	Leg*
Buch	Book
Dach	Roof
Gans	Goose
Hemd	Shirt
Holz	Wood
Hund	Dog
Kleid*	Dress*
Kopf	Head
Kuh	Cow
Obst	Fruit
Ohr	Ear
Pferd	Horse
Rauch	Smoke
Seil	Rope
Stier	Bull
Stuhl	Chair
Topf	Pot
Zahn	Tooth
Zug	Train
