# Supplementary material for: Assessing Proactive Language Control: Does Predictability of Language Sequences Benefit Language Switching?
Source: J Cogn. 2022 Apr 11;5(1):27. doi: 10.5334/joc.219 (PMC9400636; doi:10.5334/joc.219)
Supplement: Appendix B. — Overview of means per condition. [file joc-5-1-219-s2.pdf]

Appendix

B: Overview of means per condition

Table B1: Means Experiment 1 for practice effects analyses. Error rates are reported as averages across trials, where each error was coded as 1 and each accurate trial as 0. Block 7 (random sequence) is marked in gray.

			Block							
DV	Language	Language Transition	1	2	3	4	5	6	7	8
RT	L1	Repetition	1299	1286	1235	1267	1215	1233	1241	1212
		Switch	1350	1311	1341	1358	1302	1314	1247	1274
	L2	Repetition	1335	1219	1159	1188	1131	1156	1136	1161
		Switch	1377	1257	1263	1229	1233	1216	1181	1204
Error Rate	L1	Repetition	0.0247	0.0238	0.0157	0.0103	0.0210	0.0181	0.0055	0.0103
		Switch	0.0567	0.0339	0.0081	0.0194	0.0082	0.0183	0.0284	0.0135
	L2	Repetition	0.0394	0.0331	0.0289	0.0254	0.0106	0.0078	0.0163	0.0248
		Switch	0.0461	0.0426	0.0247	0.0162	0.0195	0.0213	0.0257	0.0053

Table B2: Means Experiment 1 for predictability effect analyses.

			Predictability	
DV	Language	Language Transition	Predictable	Random
RT	L1	Repetition	1227	1231
		Switch	1295	1235
	L2	Repetition	1156	1127
		Switch	1213	1183
Error Rate	L1	Repetition	0.0146	0.0058
		Switch	0.0158	0.0279
	L2	Repetition	0.0162	0.0178
		Switch	0.0133	0.0275

Table B3: Means Experiments 2-3 for practice effects analyses. Error rates are reported as averages across trials, where each error was coded as 1 and each accurate trial as 0. Block 6 (random sequence) is marked in gray.

Experiment	DV	Language	Language Transition	Block 1	2	3	4	5	6	7
Experiment 2	RT	L1	Repetition	1050	1051	1048	1036	1009	1023	1017
			Switch	1126	1090	1072	1089	1076	1071	1055
		L2	Repetition	1025	1001	1000	979	964	960	973
			Switch	1086	1046	1043	1022	1016	1021	1028
	Error Rate	L1	Repetition	0.0321	0.0154	0.0120	0.0132	0.0183	0.0049	0.0117
			Switch	0.0401	0.0561	0.0567	0.0178	0.0244	0.0259	0.0327
		L2	Repetition	0.0243	0.0049	0.0049	0.0066	0.0000	0.0033	0.0065
			Switch	0.0354	0.0215	0.0228	0.0194	0.0098	0.0227	0.0163
Experiment 3	RT	L1	Repetition	790	743	713	675	719	920	711
			Switch	923	909	906	903	920	923	873
		L2	Repetition	719	681	686	668	656	884	665
			Switch	864	849	869	881	897	924	915
	Error Rate	L1	Repetition	0.0108	0.0047	0.0061	0.0063	0.0063	0.0440	0.0139
			Switch	0.0260	0.0375	0.0301	0.0270	0.0258	0.0419	0.0244
		L2	Repetition	0.0094	0.0047	0.0049	0.0047	0.0047	0.0169	0.0032
			Switch	0.0186	0.0334	0.0422	0.0319	0.0290	0.0228	0.0337

Table B4: Means Experiments 2-3 for predictability effect analyses.

Experiment	DV	Language	Language Transition	Predictability	
				Predictable	Random
Experiment 2	RT	L1	Repetition	1012	1024
			Switch	1067	1073
		L2	Repetition	970	962
			Switch	1023	1023
	Error Rate	L1	Repetition	0.0153	0.0051
			Switch	0.0289	0.0261
		L2	Repetition	0.0033	0.0033
			Switch	0.0136	0.0224
Experiment 3	RT	L1	Repetition	713	918
			Switch	894	917
		L2	Repetition	658	883
			Switch	903	916
	Error Rate	L1	Repetition	0.0100	0.0449
			Switch	0.0258	0.0433
		L2	Repetition	0.0040	0.0178
			Switch	0.0316	0.0237
